# The Melatonin Agonist Ramelteon Induces Duration-Dependent Clock Gene Expression through cAMP Signaling in Pancreatic INS-1 β-Cells

**DOI:** 10.1371/journal.pone.0102073

**Published:** 2014-07-11

**Authors:** Keiji Nishiyama, Keisuke Hirai

**Affiliations:** CNS Drug Discovery Unit, Pharmaceutical Research Division, Takeda Pharmaceutical Company Ltd., Fujisawa, Kanagawa, Japan; Karlsruhe Institute of Technology, Germany

## Abstract

Prolonged exposure to melatonin improves glycemic control in animals. Although glucose metabolism is controlled by circadian clock genes, little is known about the role of melatonin signaling and its duration in the regulation of clock gene expression in pancreatic β-cells. Activation of MT_1_ and MT_2_ melatonin receptors inhibits cAMP signaling, which mediates clock gene expression. Therefore, this study investigated exposure duration-dependent alterations in cAMP element-binding protein (CREB) phosphorylation and clock gene expression that occur during and after exposure to ramelteon, a selective melatonin agonist used to treat insomnia. In rat INS-1 cells, a pancreatic β-cell line endogenously expressing melatonin receptors, ramelteon persistently decreased CREB phosphorylation during the treatment period (2–14 h), whereas the subsequent washout induced an enhancement of forskolin-stimulated CREB phosphorylation in a duration- and concentration-dependent manner. This augmentation was blocked by forskolin or the melatonin receptor antagonist luzindole. Similarly, gene expression analyses of 7 clock genes revealed the duration dependency of the effects of ramelteon on *Rev-erbα* and *Bmal1* expression through melatonin receptor-mediated cAMP signaling; longer exposure times (14 h) resulted in greater increases in the expression and signaling of *Rev-erbα*, which is related to β-cell functions. Interestingly, this led to amplified oscillatory *Rev-erbα* and *Bmal1* expression after agonist washout and forskolin stimulation. These results provide new insights into the duration-dependent effects of ramelteon on clock gene expression in INS-1 cells and may improve the understanding of its effect *in vivo*. The applicability of these results to pancreatic islets awaits further investigation.

## Introduction

Melatonin is a circulating hormone primarily released from the pineal gland during the night, and it is known to function as an effective chronobiotic agent capable of changing the phase and amplitude of circadian rhythms such as the sleep–wake cycle [1]. The effects of melatonin are likely exerted by activation of 2 Gi protein-coupled receptors, MT_1_ and MT_2_, leading to inhibition of cAMP production [2], [3]. In addition, activation of the MT_1_ receptor results in ERK1/2 activation [4]. The human MT_1_ receptor is expressed abundantly in the suprachiasmatic nucleus of the hypothalamus, the site of the master clock that generates circadian rhythms [5]. However, the mRNA expression of MT_1_ and MT_2_ receptors is widely detected in the human brain and peripheral tissues including the pancreatic islets [6]–[8].

Increasing evidence suggests that melatonin signaling is related to glycemic control. Prolonged exposure to melatonin through drinking water improves abnormal glucose homeostasis, such as hyperglycemia and insulinemia in rodents [9], [10]. Similarly, long-term treatment with prolonged-release melatonin exerts a beneficial effect on HbA1c levels in insomnia patients with diabetes [11]. The pancreas is considered a potential target tissue of the effect of melatonin on glucose regulation because melatonin inhibits forskolin- or high glucose-stimulated insulin secretion in both rodent islet cells and rat INS-1 cells, a pancreatic β-cell line [12], [13]. Of note, knockout studies of MT_1_ and MT_2_ receptors have demonstrated that the effects of melatonin on insulin secretion are primarily mediated via MT_1_ receptors in mouse islet cells [14].

Circadian clocks provide time cues for behavioral cycles and synchronize metabolic processes with the anticipated behavioral cycles. At the molecular level, circadian rhythms are encoded by an autoregulatory loop composed of a set of transcription activators (*Clock* and *Bmal1*) that induce the expression of repressors (*Per1–3* and *Cry1–2*) that provide feedback to inhibit the forward limb [15]. The role of circadian clocks in metabolic regulation including glucose metabolism is well supported by genetic evidence that mutations in clock genes disturb the rhythmic expression of key metabolic genes and cause metabolic disorders [16]. *Clock* mutation and *Bmal1* deficiency in mice impair glucose tolerance and insulin secretion [17]. Interestingly, the pancreatic islets of *Bmal1* knockout mice have marked defects that affect insulin exocytosis. These metabolic alterations likely reflect downstream events of core clock gene expression [16].

Previous studies revealed that melatonin directly affects clock gene expression in several types of cells [18]. However, these studies have not produced consistent results, possibly because of differences in cell types and/or experimental conditions including concentrations and exposure durations of melatonin [19]–[21]. Indeed, this duration dependency has been reported in the pars tuberalis (PT) of the pituitary, which expresses a high density of MT_1_ receptors and regulates seasonal neuroendocrine responses [22]. Of note, von Gall *et al.* demonstrated that prolonged stimulation of MT_1_ receptors in PT cells enhances cAMP signaling through the adenosine A2b receptor, leading to the rhythmic expression of PER1 [23]. This phenomenon is called sensitization; persistent activation of Gi-coupled receptors results in paradoxical activation of adenylate cyclase upon the termination of receptor-mediated inhibitory effects by ligand washout [24]. Likewise, it has been observed in several types of cells expressing MT_1_ receptors that prolonged exposure to melatonin, followed by withdrawal, potentiates forskolin-stimulated cAMP signaling [12], [25]. Furthermore, MT_1_ knockout and pinealectomized mice studies support that MT_1_ receptor-mediated melatonin signaling is crucial to circadian rhythms and the expression levels of several clock genes including *Per*1, *Cry1*, and *Rev-erbα* in PT cells [26], [27]. However, little is known about the role of melatonin signaling and its duration in clock gene expression in pancreatic β-cells. This information will facilitate the understanding of effects of melatonin and synthetic agonists for melatonin receptors on β-cell functions via clock gene expression.

This study investigated duration-dependent alterations in cAMP-mediated signaling and clock gene expression that occur during and after exposure to ramelteon, a selective MT_1_/MT_2_ agonist used to treat insomnia (Rozerem; Takeda Pharmaceutical Company, Osaka, Japan). On the basis of the aforementioned findings in PT cells, we compared the effects of brief and prolonged exposure to ramelteon on cAMP element-binding protein (CREB) phosphorylation and the expression of 7 clock genes (*Rev-erbα*, *Bmal1*, *Clock*, *Per1*, *Per2*, *Cry1*, *and Cry2*) in INS-1 β-cells endogenously expressing melatonin receptors.

## Materials and Methods

### Chemicals and drugs

Ramelteon, (*S*)-*N*-[2-(1,6,7,8-tetrahydro-2H-indeno-[5,4-b]furan-8-yl)ethyl] propionamide (TAK-375), was synthesized by Takeda Pharmaceutical Company (Osaka, Japan) [28]. H89, 2′,5′-dideoxyadenosine, and GSK4112 were purchased from Sigma-Aldrich (St. Louis, MO, USA). In addition, luzindole was obtained from Tocris Cookson (Bristol, UK). Forskolin was procured from Merck Millipore (Darmstadt, Germany).

### Cell culture

The INS-1 cell line 832/13, derived from INS-1 rat insulinoma cells, was provided by Dr. Christopher B. Newgard (Duke University Medical Center, Durham, NC, USA) [29], [30]. Cells were maintained under a 5% CO_2_/95% air atmosphere at 37°C in RPMI 1640 medium supplemented with 6 mM glucose, 1 mM sodium pyruvate, 10 mM HEPES, 10% heat-inactivated fetal bovine serum (FBS), 55 µM 2-mercaptoethanol, 100 units/mL penicillin, and 100 µg/mL streptomycin. These supplements and medium were purchased from Invitrogen (Carlsbad, CA, USA).

### In-Cell Western assay

INS-1 cells were seeded (4.7×10^4^ cells/cm^2^) in polylysine-coated 96-well plates (Sumitomo Bakelite, Tokyo, Japan) and cultured in the aforementioned growth medium for 1.5–2 days. The cells were treated with vehicle or compounds (ramelteon, H89, or 2′,5′-dideoxyadenosine in the absence or presence of luzindole or forskolin) in RPMI 1640 medium supplemented with 0.1% FBS for 2–14 h. In the sensitization experiments, the cells were subsequently washed twice with Krebs-Ringer bicarbonate-HEPES buffer containing 0.2% BSA and then incubated with the buffer for 30 min before stimulation with forskolin (0.1 µM) in the buffer for 30 min. After stimulation, the cells were fixed with 4% paraformaldehyde for 20 min at room temperature, repeatedly washed with Dulbecco's phosphate-buffered saline (Sigma-Aldrich), and incubated overnight at 4°C with rabbit anti-phospho-CREB antibody (#9198, 1∶100; Cell Signaling Technology, Danvers, MA, USA) and mouse anti-total-CREB antibody (#9104, 1∶300; Cell Signaling Technology) diluted in Dulbecco's phosphate-buffered saline containing 10% FBS and 0.1% Triton X-100. To check the specificity of phospho-CREB antibody, phospho-CREB blocking peptide (1090S, Cell Signaling Technology) was added to the antibody (a half volume of the peptide) and incubated for 1 h prior to adding it to the cells. Using Alexa Fluor 680-conjugated anti-mouse IgG (1∶200; Invitrogen) and IRDye800CW-conjugated anti-rabbit IgG (1∶800; Rockland, Gilbertsville, PA, USA) as secondary antibodies or DRAQ5 (1∶3000; Cell Signaling Technology) for nuclear staining, fluorescence signaling from each well was quantified using the Odyssey Infrared Imaging System (LI-COR, Lincoln, NE, USA).

### Insulin secretion assay

As described in the In-Cell Western assay section, INS-1 cells were pretreated with ramelteon. After 2 or 14 h treatment, the medium was collected and stored at −80°C until the insulin concentration was measured. In the sensitization and Rev-erbα agonist experiments, the cells were subsequently washed twice with Krebs-Ringer bicarbonate-HEPES buffer and incubated for 30 min in the buffer. Following stimulation of the cells with 10 µM forskolin or GSK4112 for 2 h or 30 min, respectively, insulin concentrations in the supernatants were measured using the AlphaLISA Insulin Kit (Perkin-Elmer, Wellesley, MA, USA).

### Quantitative gene expression assay

INS-1 cells were seeded (4.7×10^4^ cells/cm^2^) in polylysine-coated 24-well plates and grown for 2 days. The cells were then treated with compounds for 2–14 h, as described in the In-Cell Western assay section. The procedure for stimulation with forskolin and high glucose concentration in the oscillation experiments was as follows. The ramelteon-treated cells were washed twice and incubated with glucose- and serum-free RPMI 1640 medium for 1.5 h. The medium was then replaced with RPMI 1640 medium supplemented with high glucose (12 mM) and forskolin (0.1 µM) for 1.5 h. Next, the medium was replaced with RPMI 1640 medium supplemented with 6 mM glucose. At predetermined times (0, 1, 4, 8, 12, 16, 20, 24, 28, 32, 36, 40, 44, and 48 h), the cells were lysed with buffer RLT (Qiagen, Valencia, CA, USA) and stored at −80°C. Total RNA was extracted using the RNeasy 96 Kit and DNase I (Qiagen), according to the manufacturer's instructions. The RNA was reverse transcribed into cDNA using the High Capacity cDNA Reverse Transcription Kit (Applied Biosystems, Foster City, CA, USA), followed by quantitative real-time polymerase chain reaction using the 7900HT Sequence Detection System (Applied Biosystems). Reactions were conducted in a final volume of 20 µL containing a pair of unlabeled primers (target gene, 200 nM; cyclophilin A, 75 nM), a TaqMan probe with a FAM or HEX label (target gene, 100 nM; cyclophilin A, 16.7 nM), a cDNA sample or standard template DNA (copy numbers, 1×10^2^−1×10^7^), and quantitative real-time polymerase chain reaction MasterMix (Eurogentec, Seraing, Belgium). The primer and probe sequences are listed in Table 1. Samples of INS-1 cells were subjected to the following conditions: 2 min at 50°C; 10 min at 95°C; and 40 cycles of 15 s at 95°C and 1 min at 60°C. The amount of the target and reference genes (rat cyclophilin A) was determined using absolute quantification or relative quantification according to the ΔΔC_t_ method. Values for target mRNA expression were normalized by comparison with reference genes.

**Table 1:** Primer and probe sequences for quantitative real-time polymerase chain reaction.

Gene symbol		Non-labeled primer (5′–3′)	TaqMan probe (5′–3′)
Nr1d1	F	CAGCGAGAAGCTCAACTCTCTG	CCGTGAAAAGGCCCAGCTCCTCCTCAG
	R	CCATTCCCGAGCGGTCTGC	
Arntl	F	GGTCGAATGATTGCCGAGGAA	ACAGGATAAGAGGGTCATCACCTTCCAGC
	R	CGTACTTGTGATGTTCAGTGGG	
Clock	F	TCCCAGTCAGTTGGTTCATCATTA	CACAGCCAGCGATGTCTCAAGCTGCAA
	R	CTGAGCTGAAAGCTGAAACTGTG	
Per1	F	CAGGCTTCGTGGGCTTGAC	CCTTCAGCCCCTGGTTGCCACCATG
	R	CAGTGGTGTCGGCGACCA	
Per2	F	AGAGGTTCATCCGTGGGTCC	ACACACCCTGTTACGTCGATGGCGGTA
	R	TTGCCTTTCTCCTCACTTTCACA	
Cry1	F	TGCTCCTGGAGAGAATGTCCC	CCACTTCCTTGAGAGCAGTTTCCGCCAC
	R	TGGGTTAGTTTGCTGACTGTCTC	
Cry2	F	AGCACTTGGAACGGAAGGC	TCCGAGGTCTCTCATAGTTGGCAACCC
	R	GCCAGCAAGGAATTGGCATTC	
PGC-1α	F	GAGAGTATGAGAAGCGGGAGTC	ACACGGCGCTCTTCAATTGCTTTCTGCT
	R	GTCAGGTCTGATTTTACCAACGTAA	
Srebf1	F	CGCTCTTGACCGACATCGAA	CAACAACCAAGACAGTGACTTCCCTGGC
	R	GCCTGTGTCTCCTGTCTCAC	
Fasn	F	CGCCAGAGCCCTTTGTTAATTG	TGGGACACCCTGAGCCTGCCTCG
	R	CTAGGGATAACAGCACCTTGGTC	
Rplp0	F	GGGCATCACCACTAAAATCTCCA	ACCATTGAAATCCTGAGCGATGTGCAGCT
	R	TCCCACCTTGTCTCCAGTCTTTA	

F: forward primer, R: reverse primer.

### Western blot analysis

INS-1 cells were treated with ramelteon for 14 h and homogenized in cell extraction buffer (Invitrogen) containing a protease inhibitor cocktail (Sigma-Aldrich). Insoluble components of the lysates were removed by centrifugation at 15,000×*g* for 20 min, and protein concentrations were determined using a bicinchoninic acid protein assay kit (Pierce, Hercules, CA). The resulting lysates (10 µg of protein/lane) were subjected to sodium dodecyl sulfate-polyacrylamide gel electrophoresis using Bis-Tris-glycine gels (4–12%) and transferred to polyvinylidene difluoride membranes (Invitrogen). The membranes were probed with anti-NR1D1 antibody (AB40523, 1∶600; Abcam, Cambridge, UK) and anti-β-actin antibody (A-5441, 1∶10,000; Sigma-Aldrich) diluted with Can Get Signal Immunoreaction Enhancer solution (Toyobo, Osaka, Japan).

### Data analyses

Data shown are representative of two or more separate experiments. All statistical analyses were performed using SAS software (SAS Institute, Cary, NC, USA). Differences between the exposure durations were analyzed using 2-way analysis of variance followed by Dunnett's multiple comparisons test, and *P* values of <0.05 were considered significant. In the concentration dependence experiments, differences between the multiple dosing and control groups were assessed using a 1-tailed Williams' test, and *P* values of <0.025 were considered significant.

## Results

### Comparisons of CREB phosphorylation levels between short- and long-term treatments with ramelteon

**Figure 1:**
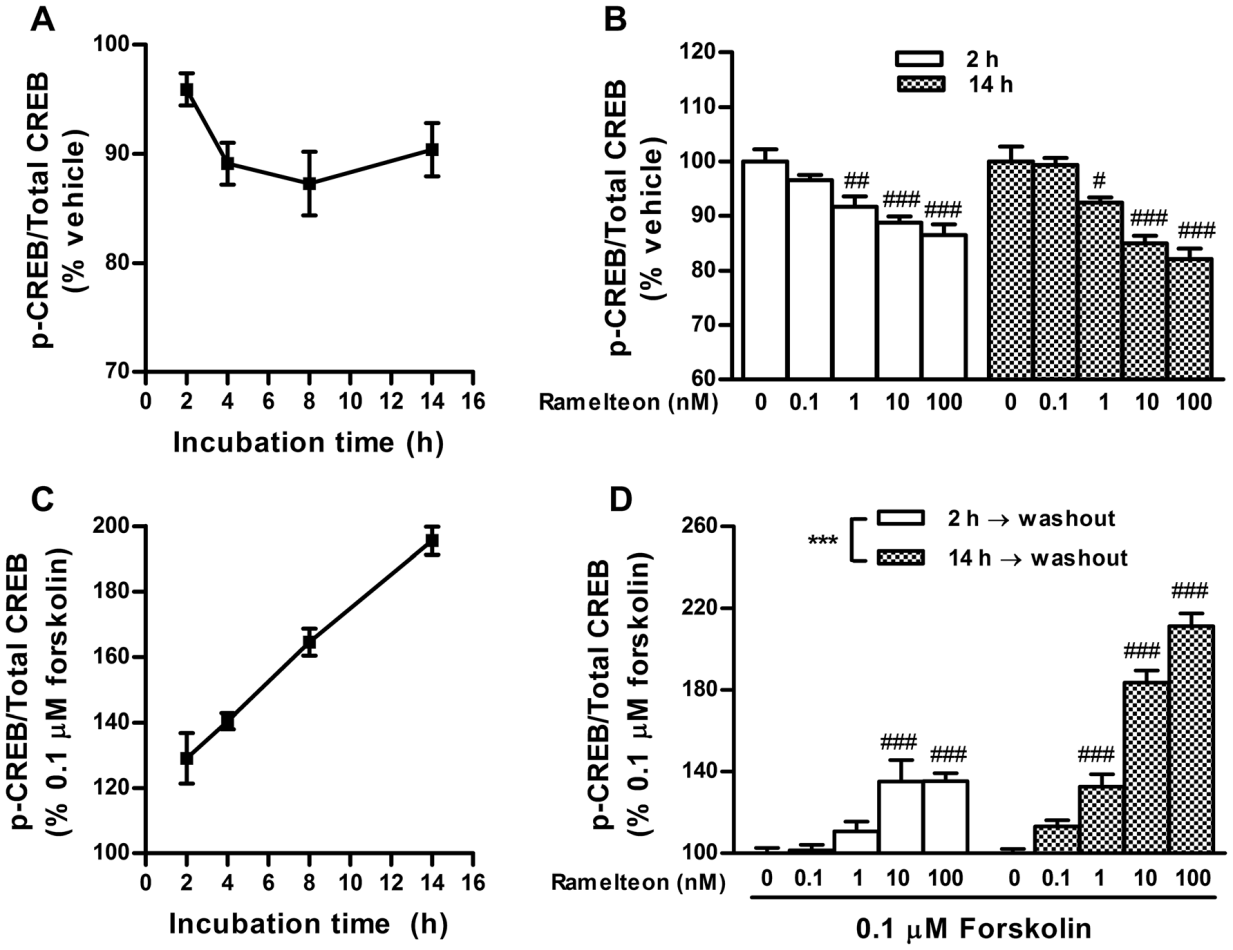
Duration-dependent changes in CREB phosphorylation during ramelteon treatment and after washout. (A) INS-1 cells were treated with ramelteon (10 nM) for 2, 4, 8, or 14 h. (B) Concentration-dependent decreases in CREB phosphorylation were assessed after ramelteon (0.1–100 nM) treatment for 2 and 14 h. (C) After ramelteon (10 nM) treatment for 2, 4, 8, or 14 h, the cells were washed twice and stimulated with forskolin (0.1 µM) for 30 min in the absence of ramelteon. (D) Concentration-dependent potentiation of forskolin-stimulated CREB phosphorylation was assessed after ramelteon (0.1–100 nM) treatment for 2 and 14 h. Values are expressed as the ratio of phosphorylated CREB to total CREB in the vehicle-pretreated control (100%). Data are presented as means ± SEM (n = 3) and were analyzed using 2-way analysis of variance followed by Dunnett's test. ***P<0.001, 2 h treatment vs. 14 h treatment; ^#^ *P*<0.05, ^##^ *P*<0.01, ^###^ *P*<0.001 vs. vehicle-pretreated control.

In this study, we used rat INS-1 cells which, like rat pancreatic islets, have been demonstrated to express both native MT_1_ and MT_2_ receptors (predominantly MT_1_ receptors) [7], [31]. Duration-dependent changes in cAMP signaling during ramelteon treatment and after washout were determined by examining the CREB (Ser133) phosphorylation level. Previous studies in INS-1 cells revealed that short-term treatment (0.5–4 h) with melatonin inhibits the cAMP signaling pathway (i.e., cAMP response element-mediated gene expression and CREB phosphorylation) [12], [31]. To further study the changes in cAMP signaling, we extended the exposure duration to 14 h. During ramelteon treatment (2–14 h), CREB phosphorylation was persistently decreased in a concentration-dependent manner, and no significant difference was observed between the 2 and 14 h treatments (Figure 1A and 1B). In contrast, washout after ramelteon treatment followed by forskolin stimulation induced sensitization, i.e., enhancement of forskolin-stimulated CREB phosphorylation (Figure 1C and 1D), as previously observed with melatonin [12]. The augmentation became more prominent with longer incubation times and higher agonist concentrations (Figure 1D). In addition, we confirmed the validity of In-Cell Western data using a blocking peptide for phospho-CREB antibody and DRAQ5 for nuclear staining (Figure S1).

### Ramelteon-induced sensitization through melatonin receptor-mediated cAMP signaling

**Figure 2:**
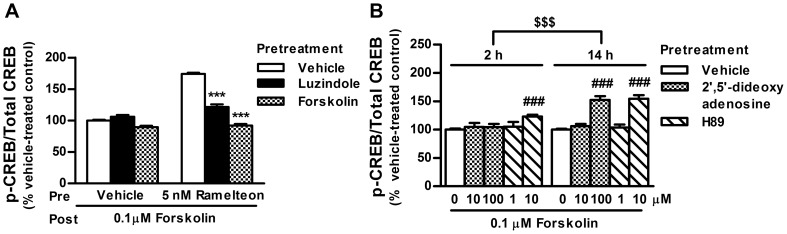
Blockade of ramelteon-induced potentiation of CREB phosphorylation by luzindole and forskolin. (A) INS-1 cells were incubated with ramelteon (5 nM) in the absence or presence of luzindole (15 µM) or forskolin (0.1 µM) for 14 h. After drug washout, the cells were subjected to a second round of forskolin stimulation (0.1 µM) for 30 min. (B) INS-1 cells were incubated with 2′,5′-dideoxyadenosine (10 or 100 µM) and H89 (1 or 10 µM) for 2 or 14 h. After drug washout, the cells were stimulated with forskolin (0.1 µM) for 30 min. Values are expressed as the ratio of phosphorylated CREB to total CREB in the vehicle-pretreated control (100%). Data are presented as means ± SEM (n = 3–6) and were analyzed using 2-way analysis of variance followed by Dunnett's test. ***P<0.001 vs. ramelteon-pretreated control; ^###^ *P*<0.001 vs. vehicle-pretreated control; ^$$$^ *P*<0.001, 2 h treatment vs. 14 h treatment.

To investigate the involvement of melatonin receptors and cAMP signaling in ramelteon-induced sensitization, ramelteon-treated cells were incubated with the melatonin receptor antagonist luzindole and forskolin. Both luzindole and forskolin blocked the sensitizing effect of ramelteon on forskolin-stimulated CREB phosphorylation (Figure 2A). Furthermore, the sensitization was mimicked by 14 h incubation with an adenylate cyclase inhibitor, 2′,5′-dideoxyadenosine, and a PKA inhibitor, H89 (Figure 2B). The sensitization became more apparent following longer incubation (14 h) with the inhibitors. Taken together, ramelteon-induced sensitization is likely attributable to inhibition of cAMP signaling via melatonin receptors.

### Comparisons of insulin secretion between short- and long-term treatments with ramelteon

**Figure 3:**
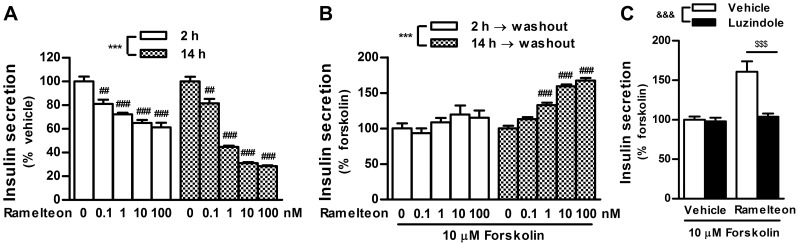
Duration-dependent changes in insulin secretion during ramelteon treatment and after drug washout. (A) INS-1 cells were treated with ramelteon (0.1–100 nM) for 2 or 14 h. (B) After ramelteon treatment for 2 or 14 h, the cells were washed twice and stimulated with forskolin (10 µM) for 2 h in the absence of ramelteon. (C) The cells were incubated with ramelteon (10 nM) in the absence or presence of luzindole (30 µM) for 14 h. After drug washout, the cells were stimulated with forskolin (10 µM) for 2 h. Values are expressed as the percentage of the vehicle- (A) or forskolin (10 µM; B and C)-stimulated insulin release in vehicle-pretreated controls. Data are presented as means ± SEM (n = 6) and were analyzed using 2-way analysis of variance followed by Dunnett's test. ***P<0.001, 2 h treatment vs. 14 h treatment; ^##^ *P*<0.01, ^###^ *P*<0.001 vs. vehicle-pretreated control; ^&&&^ *P*<0.001, luzindole group vs. vehicle group; ^$$$^ *P*<0.001 vs. ramelteon-pretreated control.

cAMP is one of the most critical messengers involved in insulin secretion. A previous report indicated that manipulation of intracellular cAMP levels in INS-1 cells by pharmacologically blocking cAMP degradation or extrusion from the cells results in increased insulin secretion [32]. To verify duration-dependent alterations in cAMP signaling at a functional level, insulin secretion from INS-1 cells was assessed using an experimental design similar to that used in the phospho-CREB analysis. Both 2 and 14 h treatments with ramelteon significantly inhibited insulin secretion (Figure 3A). However, the amount of insulin secreted after 14 h treatment was lower than that after 2 h treatment, suggesting that ramelteon continued to suppress insulin secretion beyond 2 h. Moreover, 14 h, but not 2 h, treatment, followed by washout, enhanced forskolin-stimulated insulin secretion (Figure 3B); the sensitization was blocked by luzindole (Figure 3C). Taken together with the results in the phospho-CREB analysis, these observations indicate that ramelteon can persistently inhibit cAMP signaling for a long period (14 h), resulting in sensitization after withdrawal.

### Differences in clock gene expression between short- and long-term treatments with ramelteon

**Figure 4:**
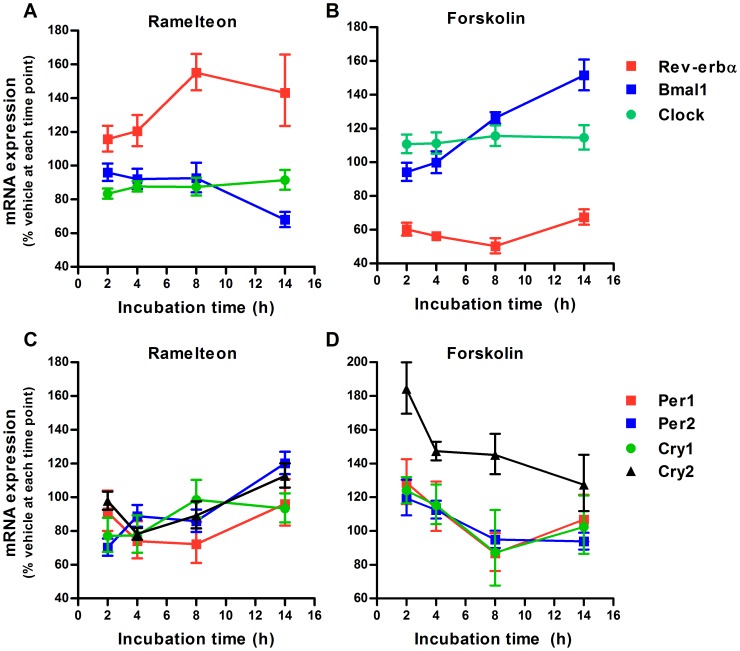
Duration-dependent changes in ramelteon-induced clock gene expression. INS-1 cells were treated with ramelteon (A and C; 10 nM) and forskolin (B and D; 0.1 µM) for 2, 4, 8, and 14 h. mRNA expression of *Rev-erbα*, *Bmal1*, *Clock*, *Per1*, *Per2*, *Cry1*, *and Cry2* was assessed using TaqMan polymerase chain reaction and normalized to that of the housekeeping gene cyclophilin A. Values are expressed as a percentage of the vehicle-treated control at each time point. Data are presented as means ± SEM (n = 4).

**Figure 5:**
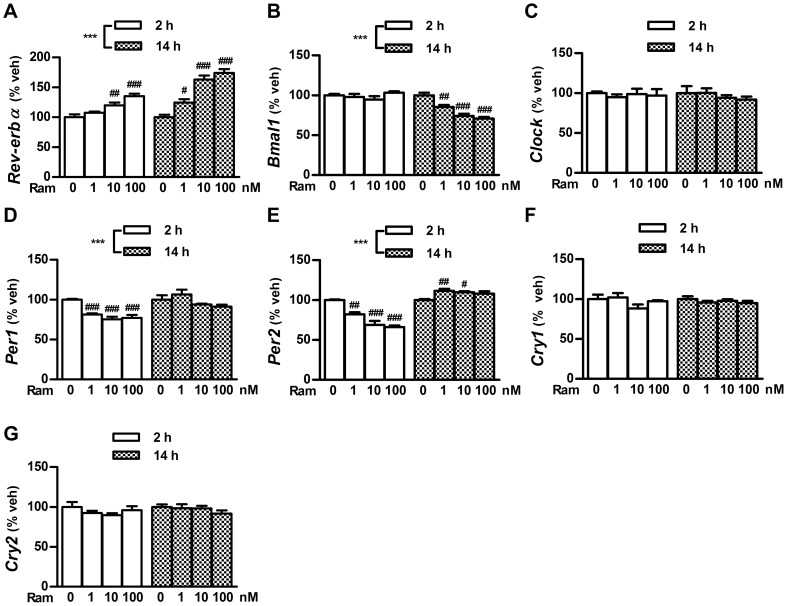
Concentration-dependent changes in ramelteon-induced clock gene expression. INS-1 cells were treated with ramelteon (1, 10, or 100 nM) for 2 or 14 h. mRNA expression of *Rev-erbα* (A), *Bmal1* (B), *Clock* (C), *Per1* (D), *Per2* (E), *Cry1* (F), and *Cry2* (G) was assessed using TaqMan polymerase chain reaction and normalized to that of the housekeeping gene cyclophilin A. Values are expressed as a percentage of the vehicle-treated control. Data are presented as means ± SEM (n = 4) and were analyzed using 2-way analysis of variance followed by Dunnett's test. ***P<0.001, 2 h treatment vs. 14 h treatment; ^#^ *P*<0.05, ^##^ *P*<0.01, ^###^ *P*<0.001 vs. vehicle-treated control.

CREB activation through the cAMP pathway is involved in the generation of circadian core clock gene expression [33], [34]. Thus, using an experimental design based on the results of phospho-CREB studies, we examined the mRNA expression of 7 clock genes (*Rev-erbα*, *Bmal1*, *Clock*, *Per1*, *Per2*, *Cry1*, *and Cry2*) in INS-1 cells after ramelteon and forskolin treatments. Ramelteon significantly activated *Rev-erbα* expression in a time- and concentration-dependent manner (Figures 4A and 5A). Consistent with the finding that Rev-erbα suppresses *Bmal1* expression [35], ramelteon gradually reduced *Bmal1* expression (Figures 4A and 5B). In contrast, *Per1* and *Per2* expression was acutely downregulated, but the magnitude of the decrease was relatively small and expression subsequently recovered within 14 h (Figures 4C, 5D, and 5E). Rather, *Per2* expression was upregulated after 14 h treatment (Figures 4C and 5E). *Clock* expression was not significantly affected by exposure of the cells to ramelteon (Figures 4A and 5C). Unlike the acute effects of melatonin in PT cells, ramelteon did not induce *Cry1* and *Cry2* expression in INS-1 cells, but rather tended to decrease them (Figure 4C, 5F and 5G) [36]. Interestingly, forskolin stimulation produced nearly opposite patterns of ramelteon-induced gene expression (Figure 4B and 4D). Therefore, ramelteon is likely to regulate the mRNA expression of particular clock genes, perhaps through the cAMP signaling pathway, in a concentration- and duration-dependent manner.

### Enhancement of Rev-erbα signaling by long-term treatment with ramelteon

**Figure 6:**
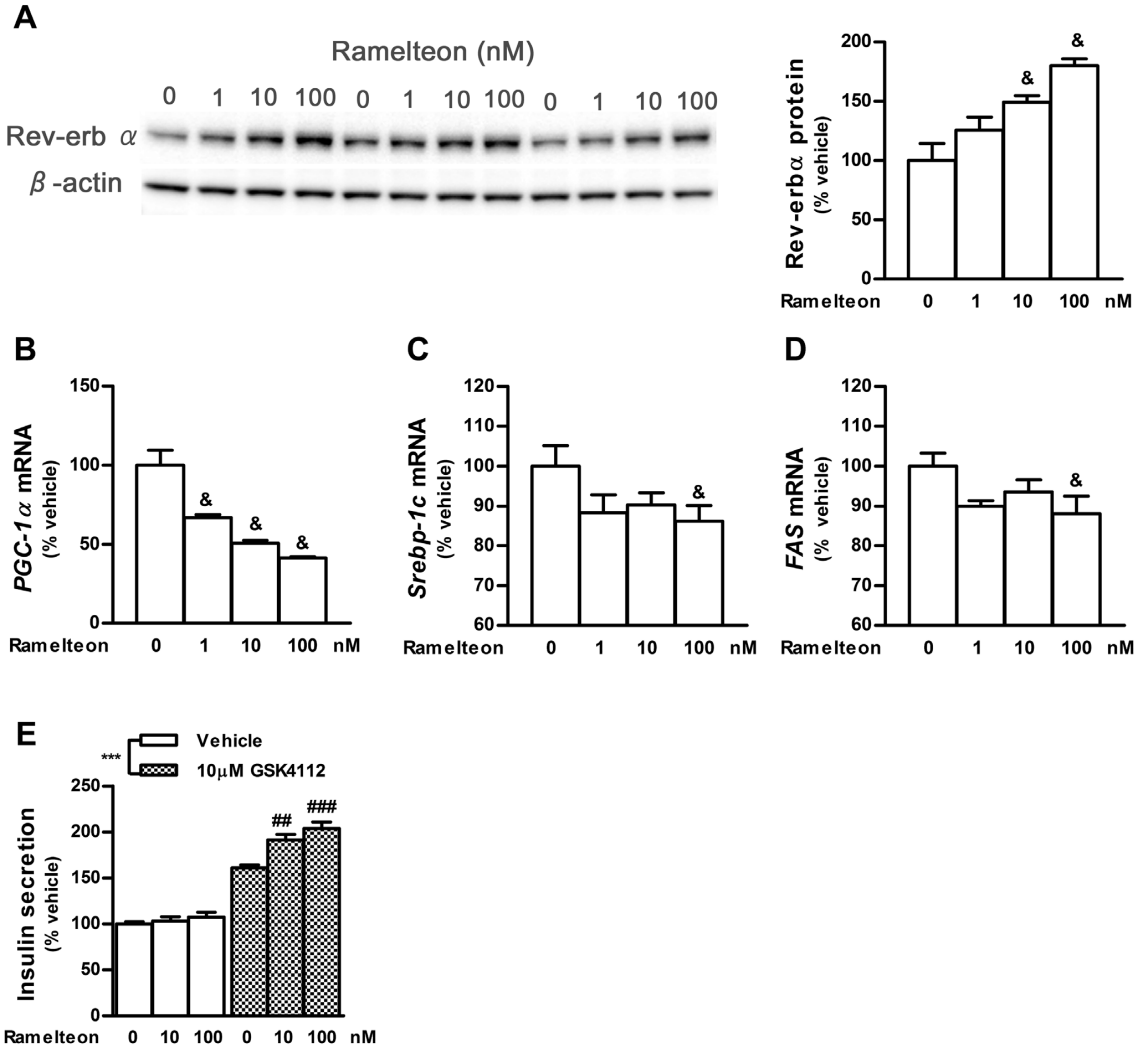
Activation of Rev-erbα signaling by ramelteon treatment for 14 h. INS-1 cells were treated with ramelteon (1, 10, or 100 nM) for 14 h. (A) Expression levels of Rev-erbα protein were detected by western blot analysis. (B–D) Expression of Rev-erbα-regulated genes (*PGC-1α*, *Srebp-1c*, and *FAS*) was measured using TaqMan polymerase chain reaction and normalized to that of the housekeeping gene cyclophilin A. (E) After ramelteon (10 or 100 nM) treatment for 14 h, the cells were washed and stimulated with the Rev-erbα agonist GSK4112 (10 µM). After 30 min, the amount of insulin secretion was measured. Data are presented as means ± SEM (A, n = 3; B–D, n = 4; E, n = 6) and were analyzed using Williams' test (A–D) or 2-way analysis of variance followed by Dunnett's test (E). ^&^ *P*<0.025 vs. vehicle-treated control; ^***^ *P*<0.001, vehicle vs. GSK4112 treatment; ^##^ *P*<0.01, ^###^ *P*<0.001 vs. GSK4112-treated control.

Rev-erbα regulates hepatic lipid metabolism involving sterol regulatory element-binding protein 1c (Srebp-1c), and Rev-erbα agonists inhibit lipid and cholesterol synthesis in the liver [16], [37]. Interestingly, recent studies illustrated that Rev-erbα controls glucagon and insulin secretion in pancreatic α- and β-cells, respectively [38], [39]. Thus, we focused on the functional activity of *Rev-erbα* after ramelteon treatment in INS-1 cells. To determine whether *Rev-erbα* mRNA upregulation by ramelteon stimulates Rev-erbα signaling, we assessed Rev-erbα protein levels and their downstream signaling in INS-1 cells after 14 h of exposure to ramelteon. Western blot analyses revealed that the 14 h treatment significantly increased Rev-erbα protein levels (Figure 6A). In accordance with findings that Rev-erbα directly represses Bmal1 transcription through the activity of a transcriptional repressor, the 14 h treatment significantly decreased *Bmal1* expression (Figure 5B) [35]. To confirm the increased ability to repress transcription, we examined the mRNA expression levels of 3 genes that were reportedly downregulated by Rev-erbα overexpression or activation: peroxisome proliferator-activated receptor γ coactivator (*PGC-1α*), *Srebp-1c*, and fatty acid synthase (*FAS*) [37], [40]. *PGC-1α* expression was markedly downregulated by ramelteon, whereas *Srebp-1c* and *FAS* expression was slightly downregulated (Figure 6B–D). Furthermore, ramelteon pretreatment facilitated insulin secretion following stimulation by the Rev-erbα agonist GSK4112 (Figure 6E). Hence, these observations indicate that long-term treatment with ramelteon can enhance Rev-erbα signaling.

### Involvement of the melatonin receptor-mediated cAMP pathway in ramelteon-induced *Rev-erbα* mRNA expression

**Figure 7:**
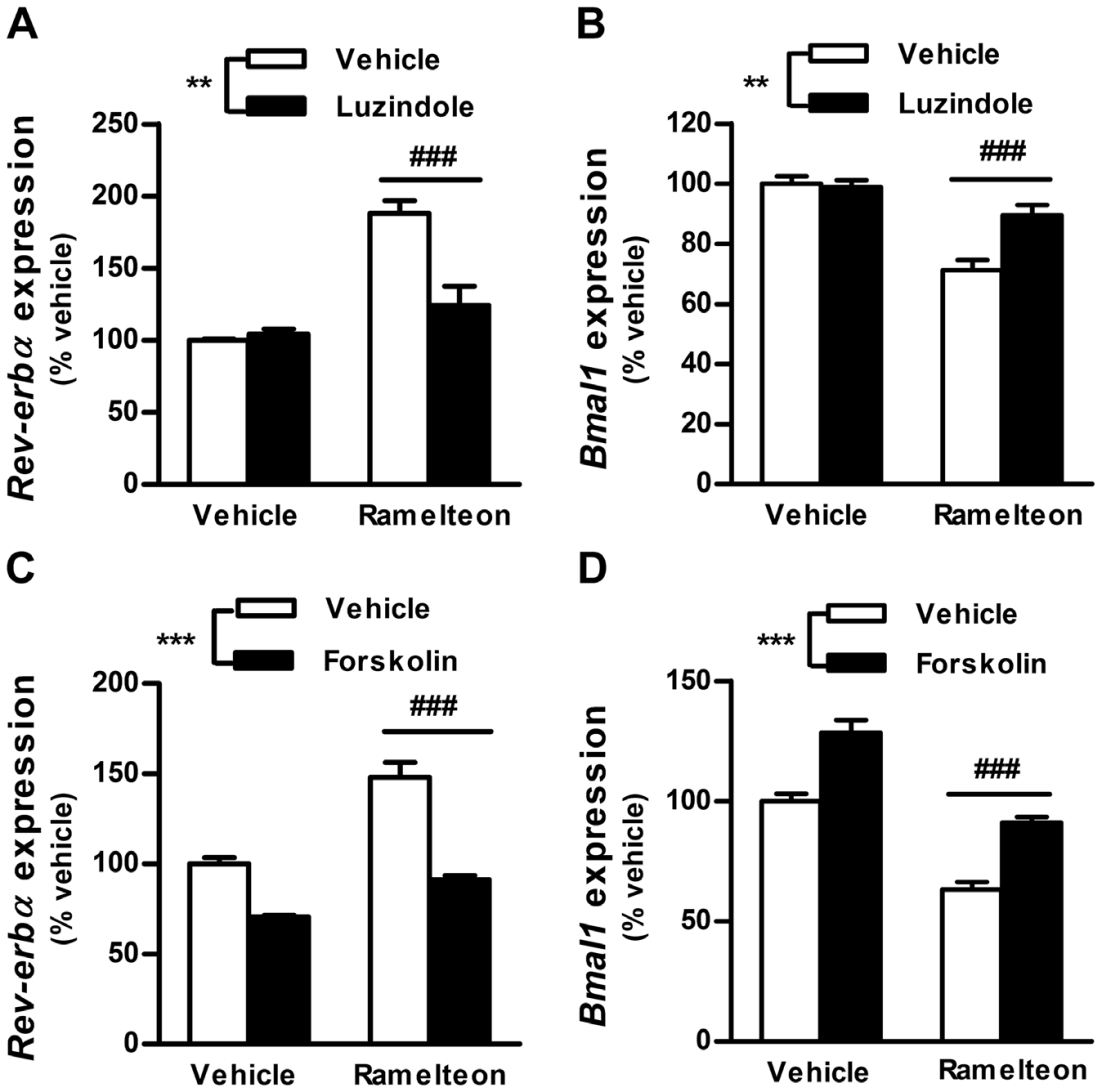
Blockade of ramelteon-induced *Rev-erbα* expression by luzindole and forskolin. INS-1 cells were incubated with ramelteon (A and B, 5 nM; C and D, 10 nM) in the absence or presence of luzindole (A and B, 15 µM) or forskolin (C and D, 0.1 µM) for 14 h. mRNA expression of *Rev-erbα* and *Bmal1* was measured using TaqMan polymerase chain reaction and normalized to that of the housekeeping gene cyclophilin A. Values are expressed as a percentage of vehicle-treated controls. Data are presented as means ± SEM (n = 4−6) and were analyzed using 2-way analysis of variance followed by Dunnett's test. ^**^ *P*<0.01, ^***^ *P*<0.001 vs. vehicle-pretreated group; ^###^ *P*<0.001 vs. ramelteon-pretreated control.

To evaluate the participation of the melatonin receptor-mediated cAMP signaling pathway in the altered mRNA expression of *Rev-erbα* and *Bmal1* by ramelteon, ramelteon-treated INS-1 cells were incubated with luzindole or forskolin for 14 h. Both luzindole and forskolin reversed ramelteon-induced alterations in *Rev-erbα* and *Bmal1* expression (Figure 7A–D), indicating that ramelteon regulates *Rev-erbα* and *Bmal1* expression through the melatonin receptor-mediated cAMP signaling pathway. In addition, the ramelteon-induced downregulation of *PGC-1α*, *Srebp-1C*, and *FAS* expression was blocked by forskolin (Figure S2).

### Oscillation in *Rev-erbα* and *Bmal1* expression after ramelteon treatment

**Figure 8:**
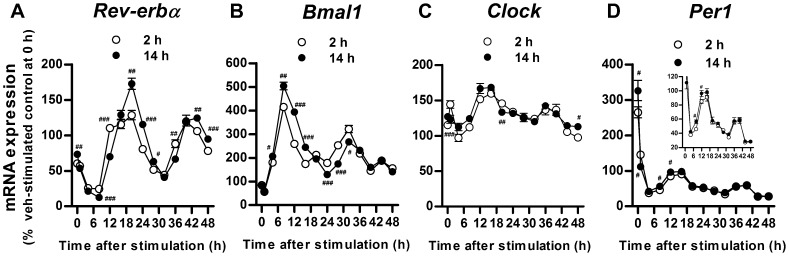
Time course of clock gene expression after ramelteon pretreatment followed by forskolin and high glucose stimulation. INS-1 cells were pretreated with ramelteon (10 nM) for 2 h or 14 h (A–D). After the washout period, the cells were stimulated with forskolin (0.1 µM) and high glucose (12 mM) for 1.5 h. Following removal of the stimulant, the cells were incubated in a serum-free medium for 0–48 h. Clock gene expression was assessed by TaqMan polymerase chain reaction and normalized to that of the housekeeping gene cyclophilin A. Values are expressed as the percentage of vehicle-stimulated controls at 0 h after stimulant removal. Data are presented as means ± SEM (n = 3) and were analyzed using 2-way analysis of variance followed by Dunnett's test. ^#^ *P*<0.05, ^##^ *P*<0.01, ^###^ *P*<0.001 vs. 2 h-pretreated control at each time point.

As described previously, in rodent pituitary cells, melatonin generates the cyclical expression of Per1, which is dependent on melatonin-induced sensitization of cAMP signaling [23]. Accordingly, ramelteon was hypothesized to generate a temporal oscillation of clock gene expression in INS-1 cells. To test this hypothesis, we monitored clock gene expression for 48 h in INS-1 cells after ramelteon (10 nM) treatment for 2 and 14 h, followed by withdrawal. On the basis of the finding that forskolin produces oscillatory clock gene expression in cultured cells, the subsequent stimulation with forskolin (0.1 µM) and high glucose (12 mM) was applied [34], [41]. Compared with 2 h pretreatment with ramelteon, 14 h pretreatment significantly enhanced the amplitude of oscillation in *Rev-erbα* and *Bmal1* expression (Figure 8A and 8B). In contrast, *Clock* expression did not display a clear difference in oscillatory pattern between treatment for 2 and 14 h (Figure 8C). Immediately after stimulation with forskolin and high glucose, induction of *Per1* expression was temporarily augmented by ramelteon treatment for 14 h compared with 2 h (Figure 8D). The concentration dependency of the ramelteon-induced expression of *Per1* and *Rev-erbα* was also observed at 0 and 9 h after forskolin stimulation, respectively (Figure S3). Thus, prolonged incubation with ramelteon appears to be essential to enhance oscillatory *Rev-erbα* and *Bmal1* expression.

## Discussion

The findings of this study provide new insights into the duration and concentration-dependent effects of ramelteon on clock gene expression in INS-1 cells. Of note, the cAMP signaling pathway predominantly contributes to alterations in clock gene expression. Ramelteon treatment for 14 h persistently inhibited cAMP signaling, as demonstrated by decreased CREB phosphorylation and reduced insulin secretion. Meanwhile, the subsequent washout induced sensitization of cAMP-mediated responses in a duration-dependent manner. Gene expression analyses of several clock genes also revealed the duration dependence of the effect of ramelteon through the cAMP signaling pathway. In particular, longer exposure times resulted in greater *Rev-erbα* expression, leading to activation of Rev-erbα signaling. Moreover, 14 h pretreatment with ramelteon, followed by washout, enhanced the oscillatory expression of *Rev-erbα* and *Bmal1* compared with the 2 h pretreatment.

Although ramelteon consistently decreased the levels of CREB phosphorylation during treatment, the extent of the decrease was quite small. Potential reasons for this small decrease include low levels of melatonin receptor expression in our cells and low activities of adenylate cyclase under our experimental conditions (a low serum concentration [0.1% FBS] and no stimulation with forskolin). In addition, higher concentrations of ramelteon were required to achieve maximum efficacy than were expected from its potency at human melatonin receptors. This might reflect a species difference in its relative potency between human and rat melatonin receptors. In fact, our preliminary data showed that the potency of ramelteon in decreasing forskolin-stimulated CREB phosphorylation in INS-1 cells was much lower than that of 2-iodomelatonin, even though their potencies at human melatonin receptors was almost the same.

In support of previous findings with melatonin, the results of our sensitization experiments indicate that the extent of ramelteon-induced sensitization of cAMP signaling is proportional to the exposure duration and concentration over wide ranges (2–14 h and 0.1–100 nM, respectively) [12], [25]. The mechanism of sensitization remains unclear; however, the ramelteon/forskolin and ramelteon/luzindole cotreatment experiments revealed that the sensitization occurs via melatonin receptor-mediated inhibition of the cAMP pathway (Figure 2A). This sensitization is likely mediated by MT_1_ receptors, together with the previous observation that MT_1_ receptor knockdown in INS-1 cells abolishes melatonin-induced sensitization [14]. Based on our results using PKA and adenylate cyclase inhibitors, PKA and/or its downstream molecules possibly contribute to ramelteon-induced sensitization (Figure 2B). Furthermore, similar to sensitization by κ-opioid receptor activation, ramelteon-induced sensitization is not probably related to receptor desensitization because pharmacological concentrations of ramelteon persistently inhibited cAMP signaling (i.e., decreased CREB phosphorylation levels and reduced insulin secretion) during long-term treatment (Figures 1 and 3) [42].

To our knowledge, this is the first study to provide evidence of the duration-dependent effects of melatonin agonists on clock gene expression in a pancreatic β-cell line (INS-1 cells). *Per1* and *Per2* mRNA expression significantly decreased after 2 h incubation with ramelteon. Subsequently, *Per1* expression recovered to control levels after 14 h treatment, whereas *Per2* expression was increased (Figures 4 and 5). The initial decreases in *Per1* and *Per2* expression were likely due to the suppression of CREB activity by ramelteon because *Per1* and *Per2* transcription is activated by CREB [33]. Given that CREB phosphorylation levels were persistently reduced during 14 h treatment, inducible cAMP early repressor reduction is hypothesized to block the suppression of *Per1* and *Per2* expression for incubation times exceeding 8 h [43]. Moreover, we found striking differences in *Rev-erbα* and *Bmal1* mRNA expression between the 2 and l4 h treatments. Interestingly, by increasing the incubation time, greater *Rev-erbα* expression was observed. This could explain the previous observation that melatonin injection modulates the phase of *Rev-erbα* expression in PT without immediate effects [44]. In addition, the increase in *Rev-erbα* expression was followed by the decrease in *Bmal1* expression. This delayed response of *Bmal1* expression might reflect the time required for *Rev-erbα* mRNA translation, resulting in repression of *Bmal1* transcription by Rev-erbα protein. *Rev-erbα* transcription is activated by Clock/Bmal1 and is transrepressed by Per/Cry, resulting in the circadian oscillation of *Rev-erbα* expression [16]. Considering both the decreased *Bmal1* expression and lack of change in *Clock* expression in our experiments, an increase in the levels of the Clock/Bmal1 complex would not be expected. Thus, although the mechanism remains unclear, forskolin, ramelteon/forskolin, and ramelteon/luzindole treatment experiments indicate that the cAMP pathway controls melatonin receptor-mediated *Rev-erbα* expression (Figures 4 and 7). Further studies are needed to elucidate the cAMP-related mechanism for regulating *Rev-erbα* expression.

Melatonin affects oscillations in *Rev-erbα* expression [27], [44], [45]. Loss of melatonin signaling by pinealectomy abolishes the rhythmicity of *Rev-erbα* expression in the rat PT but not in the suprachiasmatic nucleus [27]. This defect is partially recovered by melatonin administration through drinking water. On the other hand, studies with melatonin receptor knockout mice demonstrated that loss of MT_1_ or MT_2_ receptors increases *Rev-erbα* expression in the pancreas, in the former case in conjugation with a phase advance [46]. These findings indicate that the contribution of melatonin signaling to oscillations in *Rev-erbα* expression is considerably tissue dependent *in vivo*. The tissue dependency may result from the cell type-dependent effects of melatonin on clock gene expression *in vitro* as described previously [18]. In the present study, prolonged, but not brief, exposure of INS-1 cells to ramelteon increased the amplitude of oscillations in *Rev-erbα* and *Bmal1* expression (Figure 8). In contrast, *Per1* expression was acutely and temporally increased by prolonged exposure to ramelteon. Because forskolin stimulation as well as serum shock elicits oscillatory clock gene expression by synchronizing their expression in individual cells, ramelteon-induced sensitization of cAMP signaling may contribute to the synchronization, resulting in increased amplitude of oscillations in *Rev-erbα* and *Bmal1* expression [34], [41]. As observed in PT cells, increased *Per1* expression may also be caused by sensitization [23]. In addition, considering that *Per1/Per2* mutant mice exhibited dampened circadian *Rev-erbα* expression, the temporal induction of *Per1* potentially affects oscillatory *Rev-erbα* expression [35].

Rev-erbα, an important metabolic regulator expressed in a circadian manner in various tissues, may translate circadian signals into metabolic and inflammatory regulatory responses and vice versa [16]. Vieira *et al.* revealed that *Rev-erbα* downregulation by siRNA in pancreatic islet and MIN-6 cells impairs β-cell functions, including glucose-induced insulin release and cell proliferation [38]. Thus, the induction of *Rev-erbα* expression by ramelteon was hypothesized to influence β-cell functions. In accordance with this hypothesis, ramelteon exposure enhanced Rev-erbα agonist-stimulated insulin release and decreased the expression of the *Rev-erbα*-regulated genes *Bmal1*, *PGC-1α*, *Srebp-1c*, and *FAS* (Figures 5 and 6). Indirect regulation of *Srebp-1c* and *FAS* expression by Rev-erbα might explain the smaller decrease in the expression of these genes than of *Bmal1* and *PGC-1α*. Intriguingly, it has been proposed that acute induction of *Cry1* expression and sensitization of *Per1* expression by melatonin in PT have significant roles in decoding the duration of melatonin signaling and regulating thyrotrophin-stimulating hormone expression, a key output hormone of PT [22]. Similarly, Rev-erbα might translate the duration of melatonin signaling into β-cell responses.

In the present study, we used INS-1 cells as a model of pancreatic islets on the basis of their similar responses to melatonin (e.g. sensitization to forskolin, inhibition of insulin secretion, etc.) [12]–[14]. However, these cells might differ in their regulation of clock gene expression. Possible supporting this, the relative mRNA expression levels of clock genes in INS-1 cells differed from those in rodent islets (Figure S4): while *Per1* and *Clock* were most expressed in INS-1 cells, *Rev-erbα* and *Clock* were more highly expressed in mouse islets [38]. Further investigation of alterations in clock gene expression in pancreatic islets is particularly required.

In conclusion, our data emphasize the importance of the duration of melatonin signaling in regulating clock gene expression in INS-1 cells, as previously reported in PT cells (Figure S5). Our results may aid in designing experiments to test the duration-dependent effects of melatonin agonists *in vivo* and understanding these effects. The applicability of these results to pancreatic islets requires further investigation.

## Supporting Information

Figure S1 **Confirmation of In-Cell Western data on p-CREB/Total CREB using a p-CREB blocking peptide and DRAQ5.** After ramelteon (10 nM) treatment for 14 h, the cells were washed twice and stimulated with vehicle or forskolin (0.1 µM) for 30 min in the absence of ramelteon. To check the specificity of phospho-CREB antibody, phospho-CREB blocking peptide was add to the antibody and incubated for 1 h prior to adding it to the cells. DRAQ5 was used for normalization of cell numbers instead of total CREB antibody. Values are expressed as the ratio of phosphorylated CREB to total CREB. Data are presented as means ± SEM (n = 4−5).
(TIF)

Figure S2 **Blockade of ramelteon-induced** ***PGC-1α*** **,** ***Srebp-1c*** **, and** ***FAS*** **expression by forskolin.** INS-1 cells were incubated with ramelteon (10 nM) in the absence or presence of forskolin (0.1 µM) for 14 h. mRNA expressions of *PGC-1α*, *Srebp-1c*, and *FAS* were measured using TaqMan polymerase chain reaction and normalized to that of the housekeeping gene cyclophilin A. Values are expressed as a percentage of the vehicle-treated controls. Data were analyzed using 2-way analysis of variance followed by Dunnett's test and are presented as means ± SEM (n = 4−6). ^***^ *P*<0.001 vs. vehicle-pretreated group; ^##^ *P*<0.01, ^###^ *P*<0.001 vs. ramelteon-pretreated control.
(TIF)

Figure S3 **Concentration-dependent alterations in** ***Per1*** **and** ***Rev-erbα*** **expression after ramelteon pretreatment followed by forskolin and high glucose stimulation.** INS-1 cells were pretreated with ramelteon (10 nM) for 14 h. After the washout period, the cells were stimulated with forskolin (0.1 µM) and a high concentration of glucose (12 mM) for 1.5 h. Following removal of the stimulant, the cells were incubated in a serum-free medium for 0 (A) or 9 h (B). Clock gene expression was assessed by TaqMan polymerase chain reaction and normalized to that of the housekeeping gene cyclophilin A. Values are expressed as a percentage of vehicle-stimulated controls at 0 (A) or 9 h (B) after stimulant removal. Data were analyzed using Williams' test and are presented as means ± SEM (n = 4−6).
(TIF)

Figure S4 **Comparative expression of clock genes in INS-1 cells.** Clock gene expression was assessed by TaqMan polymerase chain reaction and normalized to that of the housekeeping gene cyclophilin A. Values are expressed as a percentage of *Rev-erbα* expression. Data are presented as means ± SEM (n = 4).
(TIF)

Figure S5 **A schematic diagram of ramelteon signaling pathway dependent on the exposure duration in INS-1 cells.**
(TIF)

## References

[1] Arendt J, Skene DJ (2005) Melatonin as a chronobiotic. Sleep Med Rev 9: 25–39. 15649736 10.1016/j.smrv.2004.05.002

[2] Reppert SM, Weaver DR, Ebisawa T (1994) Cloning and characterization of a mammalian melatonin receptor that mediates reproductive and circadian responses. Neuron 13: 1177–1185. 7946354 10.1016/0896-6273(94)90055-8

[3] Reppert SM, Godson C, Mahle CD, Weaver DR, Slaugenhaupt SA, et al (1995) Molecular characterization of a second melatonin receptor expressed in human retina and brain: the Mel1b melatonin receptor. Proc Natl Acad Sci U S A 92: 8734–8738. 7568007 10.1073/pnas.92.19.8734 PMC41041

[4] Witt-Enderby PA, MacKenzie RS, McKeon RM, Carroll EA, Bordt SL, et al (2000) Melatonin induction of filamentous structures in non-neuronal cells that is dependent on expression of the human mt1 melatonin receptor. Cell Motil Cytoskeleton 46: 28–42. 10842331 10.1002/(SICI)1097-0169(200005)46:1<28::AID-CM4>3.0.CO;2-5

[5] Weaver DR, Reppert SM (1996) The Mel1a melatonin receptor gene is expressed in human suprachiasmatic nuclei. Neuroreport 8: 109–112. 9051762 10.1097/00001756-199612200-00022

[6] Dubocovich ML, Delagrange P, Krause DN, Sugden D, Cardinali DP, et al (2010) International Union of Basic and Clinical Pharmacology. LXXV. Nomenclature, classification, and pharmacology of G protein-coupled melatonin receptors. Pharmacol Rev 62: 343–380. 20605968 10.1124/pr.110.002832 PMC2964901

[7] Muhlbauer E, Peschke E (2007) Evidence for the expression of both the MT1- and in addition, the MT2-melatonin receptor, in the rat pancreas, islet and beta-cell. J Pineal Res 42: 105–106. 17198545 10.1111/j.1600-079X.2006.00399.x

[8] Ramracheya RD, Muller DS, Squires PE, Brereton H, Sugden D, et al (2008) Function and expression of melatonin receptors on human pancreatic islets. J Pineal Res 44: 273–279. 18194202 10.1111/j.1600-079X.2007.00523.x

[9] Rasmussen DD, Boldt BM, Wilkinson CW, Yellon SM, Matsumoto AM (1999) Daily melatonin administration at middle age suppresses male rat visceral fat, plasma leptin, and plasma insulin to youthful levels. Endocrinology 140: 1009–1012. 9927336 10.1210/endo.140.2.6674

[10] Agil A, Rosado I, Ruiz R, Figueroa A, Zen N, et al (2012) Melatonin improves glucose homeostasis in young Zucker diabetic fatty rats. J Pineal Res 52: 203–210. 21883445 10.1111/j.1600-079X.2011.00928.x

[11] Garfinkel D, Zorin M, Wainstein J, Matas Z, Laudon M, et al (2011) Efficacy and safety of prolonged-release melatonin in insomnia patients with diabetes: a randomized, double-blind, crossover study. Diabetes Metab Syndr Obes 4: 307–313. 21887103 10.2147/DMSO.S23904 PMC3160855

[12] Kemp DM, Ubeda M, Habener JF (2002) Identification and functional characterization of melatonin Mel 1a receptors in pancreatic beta cells: potential role in incretin-mediated cell function by sensitization of cAMP signaling. Mol Cell Endocrinol 191: 157–166. 12062899 10.1016/s0303-7207(02)00064-3

[13] Picinato MC, Haber EP, Cipolla-Neto J, Curi R, de Oliveira Carvalho CR, et al (2002) Melatonin inhibits insulin secretion and decreases PKA levels without interfering with glucose metabolism in rat pancreatic islets. J Pineal Res 33: 156–160. 12220330 10.1034/j.1600-079x.2002.02903.x

[14] Muhlbauer E, Albrecht E, Bazwinsky-Wutschke I, Peschke E (2012) Melatonin influences insulin secretion primarily via MT(1) receptors in rat insulinoma cells (INS-1) and mouse pancreatic islets. J Pineal Res 52: 446–459. 22288848 10.1111/j.1600-079X.2012.00959.x

[15] Takahashi JS, Hong HK, Ko CH, McDearmon EL (2008) The genetics of mammalian circadian order and disorder: implications for physiology and disease. Nat Rev Genet 9: 764–775. 18802415 10.1038/nrg2430 PMC3758473

[16] Bass J, Takahashi JS (2010) Circadian integration of metabolism and energetics. Science 330: 1349–1354. 21127246 10.1126/science.1195027 PMC3756146

[17] Marcheva B, Ramsey KM, Buhr ED, Kobayashi Y, Su H, et al (2010) Disruption of the clock components CLOCK and BMAL1 leads to hypoinsulinaemia and diabetes. Nature 466: 627–631. 20562852 10.1038/nature09253 PMC2920067

[18] Hardeland R, Madrid JA, Tan DX, Reiter RJ (2012) Melatonin, the circadian multioscillator system and health: the need for detailed analyses of peripheral melatonin signaling. J Pineal Res 52: 139–166. 22034907 10.1111/j.1600-079X.2011.00934.x

[19] Imbesi M, Arslan AD, Yildiz S, Sharma R, Gavin D, et al (2009) The melatonin receptor MT1 is required for the differential regulatory actions of melatonin on neuronal 'clock' gene expression in striatal neurons in vitro. J Pineal Res 46: 87–94. 18798788 10.1111/j.1600-079X.2008.00634.x

[20] Xiang S, Mao L, Duplessis T, Yuan L, Dauchy R, et al (2012) Oscillation of clock and clock controlled genes induced by serum shock in human breast epithelial and breast cancer cells: regulation by melatonin. Breast Cancer (Auckl) 6: 137–150. 23012497 10.4137/BCBCR.S9673 PMC3448497

[21] Alonso-Vale MI, Andreotti S, Mukai PY, Borges-Silva C, Peres SB, et al (2008) Melatonin and the circadian entrainment of metabolic and hormonal activities in primary isolated adipocytes. J Pineal Res 45: 422–429. 18662218 10.1111/j.1600-079X.2008.00610.x

[22] Barrett P, Bolborea M (2012) Molecular pathways involved in seasonal body weight and reproductive responses governed by melatonin. J Pineal Res 52: 376–388. 22017374 10.1111/j.1600-079X.2011.00963.x

[23] von Gall C, Garabette ML, Kell CA, Frenzel S, Dehghani F, et al (2002) Rhythmic gene expression in pituitary depends on heterologous sensitization by the neurohormone melatonin. Nat Neurosci 5: 234–238. 11836530 10.1038/nn806

[24] Watts VJ, Neve KA (2005) Sensitization of adenylate cyclase by Galpha i/o-coupled receptors. Pharmacol Ther 106: 405–421. 15922020 10.1016/j.pharmthera.2004.12.005

[25] Witt-Enderby PA, Masana MI, Dubocovich ML (1998) Physiological exposure to melatonin supersensitizes the cyclic adenosine 3',5'-monophosphate-dependent signal transduction cascade in Chinese hamster ovary cells expressing the human mt1 melatonin receptor. Endocrinology 139: 3064–3071. 9645677 10.1210/endo.139.7.6102

[26] von Gall C, Weaver DR, Moek J, Jilg A, Stehle JH, et al (2005) Melatonin plays a crucial role in the regulation of rhythmic clock gene expression in the mouse pars tuberalis. Ann N Y Acad Sci 1040: 508–511. 15891103 10.1196/annals.1327.105

[27] Agez L, Laurent V, Guerrero HY, Pevet P, Masson-Pevet M, et al (2009) Endogenous melatonin provides an effective circadian message to both the suprachiasmatic nuclei and the pars tuberalis of the rat. J Pineal Res 46: 95–105. 19090912 10.1111/j.1600-079X.2008.00636.x

[28] Uchikawa O, Fukatsu K, Tokunoh R, Kawada M, Matsumoto K, et al (2002) Synthesis of a novel series of tricyclic indan derivatives as melatonin receptor agonists. J Med Chem 45: 4222–4239. 12213063 10.1021/jm0201159

[29] Hohmeier HE, Mulder H, Chen G, Henkel-Rieger R, Prentki M, et al (2000) Isolation of INS-1-derived cell lines with robust ATP-sensitive K+ channel-dependent and -independent glucose-stimulated insulin secretion. Diabetes 49: 424–430. 10868964 10.2337/diabetes.49.3.424

[30] Tsujihata Y, Ito R, Suzuki M, Harada A, Negoro N, et al (2011) TAK-875, an orally available G protein-coupled receptor 40/free fatty acid receptor 1 agonist, enhances glucose-dependent insulin secretion and improves both postprandial and fasting hyperglycemia in type 2 diabetic rats. J Pharmacol Exp Ther 339: 228–237. 21752941 10.1124/jpet.111.183772

[31] Bazwinsky-Wutschke I, Wolgast S, Muhlbauer E, Albrecht E, Peschke E (2012) Phosphorylation of cyclic AMP-response element-binding protein (CREB) is influenced by melatonin treatment in pancreatic rat insulinoma beta-cells (INS-1). J Pineal Res 53: 344–357. 22616931 10.1111/j.1600-079X.2012.01004.x

[32] Peschke E, Muhlbauer E, Musshoff U, Csernus VJ, Chankiewitz E, et al (2002) Receptor (MT(1)) mediated influence of melatonin on cAMP concentration and insulin secretion of rat insulinoma cells INS-1. J Pineal Res 33: 63–71. 12153439 10.1034/j.1600-079x.2002.02919.x

[33] Travnickova-Bendova Z, Cermakian N, Reppert SM, Sassone-Corsi P (2002) Bimodal regulation of mPeriod promoters by CREB-dependent signaling and CLOCK/BMAL1 activity. Proc Natl Acad Sci U S A 99: 7728–7733. 12032351 10.1073/pnas.102075599 PMC124335

[34] Yagita K, Okamura H (2000) Forskolin induces circadian gene expression of rPer1, rPer2 and dbp in mammalian rat-1 fibroblasts. FEBS Lett 465: 79–82. 10620710 10.1016/s0014-5793(99)01724-x

[35] Preitner N, Damiola F, Lopez-Molina L, Zakany J, Duboule D, et al (2002) The orphan nuclear receptor REV-ERBalpha controls circadian transcription within the positive limb of the mammalian circadian oscillator. Cell 110: 251–260. 12150932 10.1016/s0092-8674(02)00825-5

[36] Dardente H, Menet JS, Poirel VJ, Streicher D, Gauer F, et al (2003) Melatonin induces Cry1 expression in the pars tuberalis of the rat. Brain Res Mol Brain Res 114: 101–106. 12829319 10.1016/s0169-328x(03)00134-7

[37] Solt LA, Wang Y, Banerjee S, Hughes T, Kojetin DJ, et al (2012) Regulation of circadian behaviour and metabolism by synthetic REV-ERB agonists. Nature 485: 62–68. 22460951 10.1038/nature11030 PMC3343186

[38] Vieira E, Marroqui L, Batista TM, Caballero-Garrido E, Carneiro EM, et al (2012) The clock gene Rev-erbalpha regulates pancreatic beta-cell function: modulation by leptin and high-fat diet. Endocrinology 153: 592–601. 22166979 10.1210/en.2011-1595

[39] Vieira E, Marroqui L, Figueroa AL, Merino B, Fernandez-Ruiz R, et al (2013) Involvement of the clock gene Rev-erb alpha in the regulation of glucagon secretion in pancreatic alpha-cells. PLoS One 8: e69939. 23936124 10.1371/journal.pone.0069939 PMC3723646

[40] Wu N, Yin L, Hanniman EA, Joshi S, Lazar MA (2009) Negative feedback maintenance of heme homeostasis by its receptor, Rev-erbalpha. Genes Dev 23: 2201–2209. 19710360 10.1101/gad.1825809 PMC2751986

[41] Balsalobre A, Marcacci L, Schibler U (2000) Multiple signaling pathways elicit circadian gene expression in cultured Rat-1 fibroblasts. Curr Biol 10: 1291–1294. 11069111 10.1016/s0960-9822(00)00758-2

[42] Li JG, Zhang F, Jin XL, Liu-Chen LY (2003) Differential regulation of the human kappa opioid receptor by agonists: etorphine and levorphanol reduced dynorphin A- and U50,488H-induced internalization and phosphorylation. J Pharmacol Exp Ther 305: 531–540. 12606694 10.1124/jpet.102.045559

[43] Schwartz WJ, Aronin N, Sassone-Corsi P (2005) Photoinducible and rhythmic ICER-CREM immunoreactivity in the rat suprachiasmatic nucleus. Neurosci Lett 385: 87–91. 15936880 10.1016/j.neulet.2005.05.018

[44] Wagner GC, Johnston JD, Tournier BB, Ebling FJ, Hazlerigg DG (2007) Melatonin induces gene-specific effects on rhythmic mRNA expression in the pars tuberalis of the Siberian hamster (Phodopus sungorus). Eur J Neurosci 25: 485–490. 17284190 10.1111/j.1460-9568.2006.05291.x

[45] Agez L, Laurent V, Pevet P, Masson-Pevet M, Gauer F (2007) Melatonin affects nuclear orphan receptors mRNA in the rat suprachiasmatic nuclei. Neuroscience 144: 522–530. 17067745 10.1016/j.neuroscience.2006.09.030

[46] Muhlbauer E, Gross E, Labucay K, Wolgast S, Peschke E (2009) Loss of melatonin signalling and its impact on circadian rhythms in mouse organs regulating blood glucose. Eur J Pharmacol 606: 61–71. 19374844 10.1016/j.ejphar.2009.01.029

