# Supplementary figures and images for: The Melatonin Agonist Ramelteon Induces Duration-Dependent Clock Gene Expression through cAMP Signaling in Pancreatic INS-1 β-Cells
Source: PLoS One. 2014 Jul 11;9(7):e102073. doi: 10.1371/journal.pone.0102073 (PMC4094524; doi:10.1371/journal.pone.0102073)

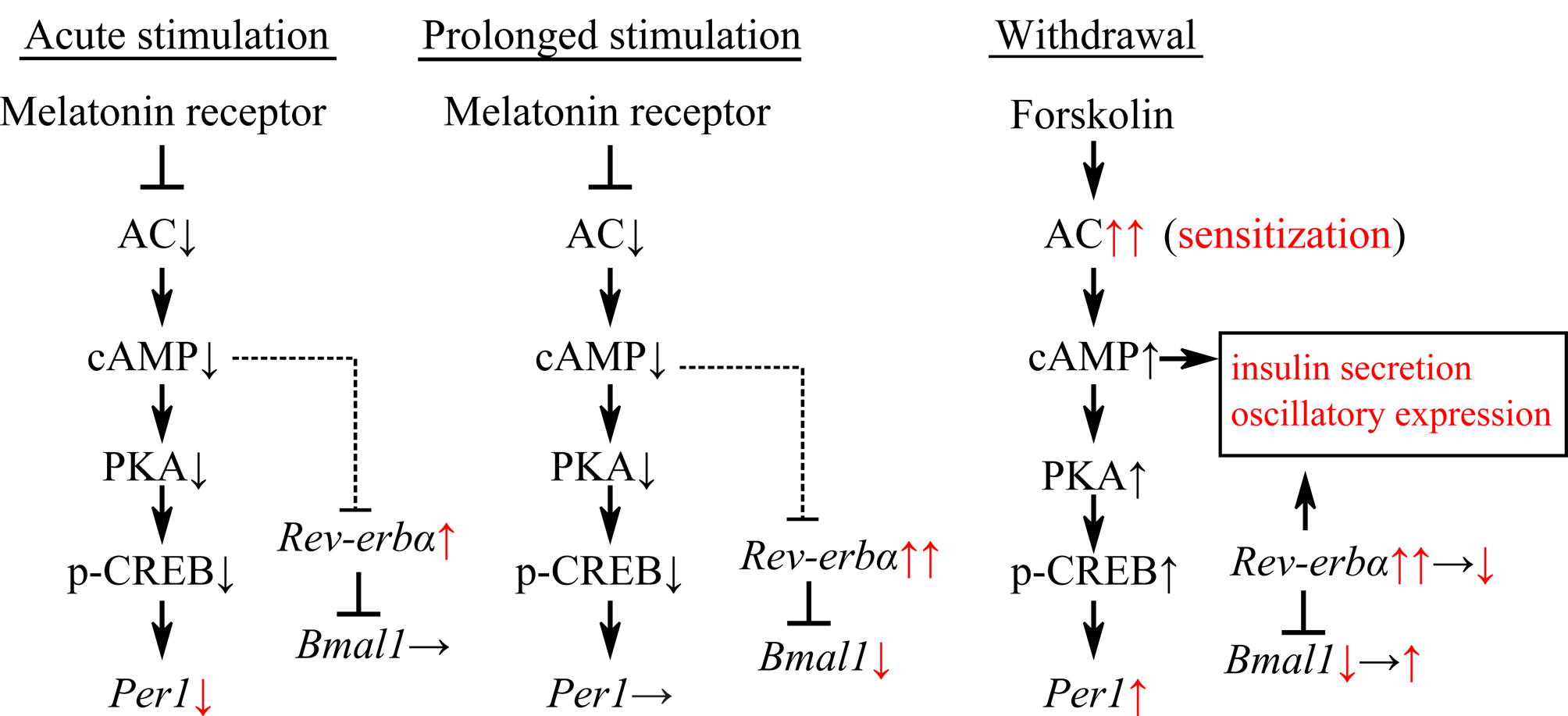

Supplement: Figure S5 — A schematic diagram of ramelteon signaling pathway dependent on the exposure duration in INS-1 cells. (TIF) [file pone.0102073.s005.tif]
